# Celiac disease in the Mediterranean area

**DOI:** 10.1186/1471-230X-14-24

**Published:** 2014-02-11

**Authors:** Francesca Tucci, Luca Astarita, Abdelhak Abkari, Mona Abu-Zekry, Thomas Attard, Mongi Ben Hariz, José Ramon Bilbao, Ghazalia Boudraa, Samir Boukthir, Stefano Costa, Veselinka Djurisic, Jean-Pierre Hugot, Iñaki Irastorza, Aydan Kansu, Sanja Kolaček, Giuseppe Magazzù, Dušanka Mičetić-Turk, Zrinjka Misak, Eleftheria Roma, Pasqualino Rossi, Selma Terzic, Virtut Velmishi, Carmela Arcidiaco, Renata Auricchio, Luigi Greco

**Affiliations:** 1 European Laboratory for Food Induced Diseases, University of Naples Federico II, Naples 80131, Italy; 2 Hôpital des Enfant IbnRochd de Casablanca, Casablanca 20050, Morocco; 3 Children’s Hospital, Gastrointestinal Unit, Cairo University, Cairo 12511, Egypt; 4 Mater Dei Hospital, Msida MSD 2090, Malta; 5 Paediatric Unit, Mongi SLIM’s Hospital of Tunis, Marsa 2078, Tunisia; 6 Endocrinology, Diabetes and Nutrition Research Group, Hospital de Cruces, Barakaldo-Bizkaia, Basque Country 48903, Spain; 7 Clinique Amilcar Cabral, Oran 31026, Algeria; 8 Hôpital d’enfants, Tunis, Tunisia; 9 Regional Celiac Center, University Hospital G. Martino, Messina 98125, Italy; 10 Clinical Centre of Montenegro, Institute for Children s Disease, Podgorica, Montenegro; 11 UMR843, INSERM, Assistance Publique Hopitaux de Paris et Université, Paris Diderot, Paris, France; 12 Pediatric Gastroenterology, Hospital de Cruces, Barakaldo 48903, Spain; 13 Faculty of Medicine, Department of Pediatric Gastroenterology, Ankara University, Ankara 06100, Turkey; 14 Children’s Hospital Zagreb, Zagreb 10000, Croatia; 15 University Medical Centre Paediatric Department, Ljubljanska, Maribor 2000, Slovenia; 16 Aghia Sophia Children’s Hospital, Athens University, Goudi, Athens 11527, Greece; 17 International Affairs Direction, Ministry of Health, Rome, Italy; 18 Department of Children Diseases, University Clinical Center, Tuzla, Bosnia Herzegovina; 19 Gastrohepatology of University Hospital Centre “Mother Teresa”, 1000 Tirana, Albania; 20 European Laboratory for Food-Induced Diseases, Department of Translational Medical Science, Section of Pediatrics, University of Naples Federico II, Via Sergio Pansini 5, 80131 Naples, Italy; 21 BioCruces Research Institute, Cruces University Hospital, University of the Basque Country (UPV-EHU), Barakaldo, Basque Country, Spain

**Keywords:** Mediterranean area, Celiac disease, World gastroenterology organization, ESPGHAN guidelines

## Abstract

**Background:**

The World Gastroenterology Organization recommends developing national guidelines for the diagnosis of Celiac Disease (CD): hence a profile of the diagnosis of CD in each country is required. We aim to describe a cross-sectional picture of the clinical features and diagnostic facilities in 16 countries of the Mediterranean basin. Since a new ESPGHAN diagnostic protocol was recently published, our secondary aim is to estimate how many cases in the same area could be identified without a small intestinal biopsy.

**Methods:**

By a stratified cross-sectional retrospective study design, we examined clinical, histological and laboratory data from 749 consecutive unselected CD children diagnosed by national referral centers.

**Results:**

The vast majority of cases were diagnosed before the age of 10 (median: 5 years), affected by diarrhea, weight loss and food refusal, as expected. Only 59 cases (7.8%) did not suffer of major complaints. Tissue transglutaminase (tTG) assay was available, but one-third of centers reported financial constraints in the regular purchase of the assay kits. 252 cases (33.6%) showed tTG values over 10 times the local normal limit. Endomysial antibodies and HLA typing were routinely available in only half of the centers. CD was mainly diagnosed from small intestinal biopsy, available in all centers. Based on these data, only 154/749 cases (20.5%) would have qualified for a diagnosis of CD without a small intestinal biopsy, according to the new ESPGHAN protocol.

**Conclusions:**

This cross-sectional study of CD in the Mediterranean referral centers offers a puzzling picture of the capacities to deal with the emerging epidemic of CD in the area, giving a substantive support to the World Gastroenterology Organization guidelines.

## Background

Celiac disease (CD) increased at an unexpected rate in the last two decades [1-3]. It was long considered a problem limited to the wheat-consuming affluent societies of the western world [4], but it was recently reported in almost every wheat-consuming country worldwide [5]. In the near future, most CD cases are expected to come from Africa, Asia and South America [6-9]. We estimate that more than 5 million cases will occur in the Mediterranean region in the next 10 years. Without a timely diagnosis and appropriate health care, an excess mortality of more than 230,000 cases may be expected in the next decade [10].

**Figure 1:**
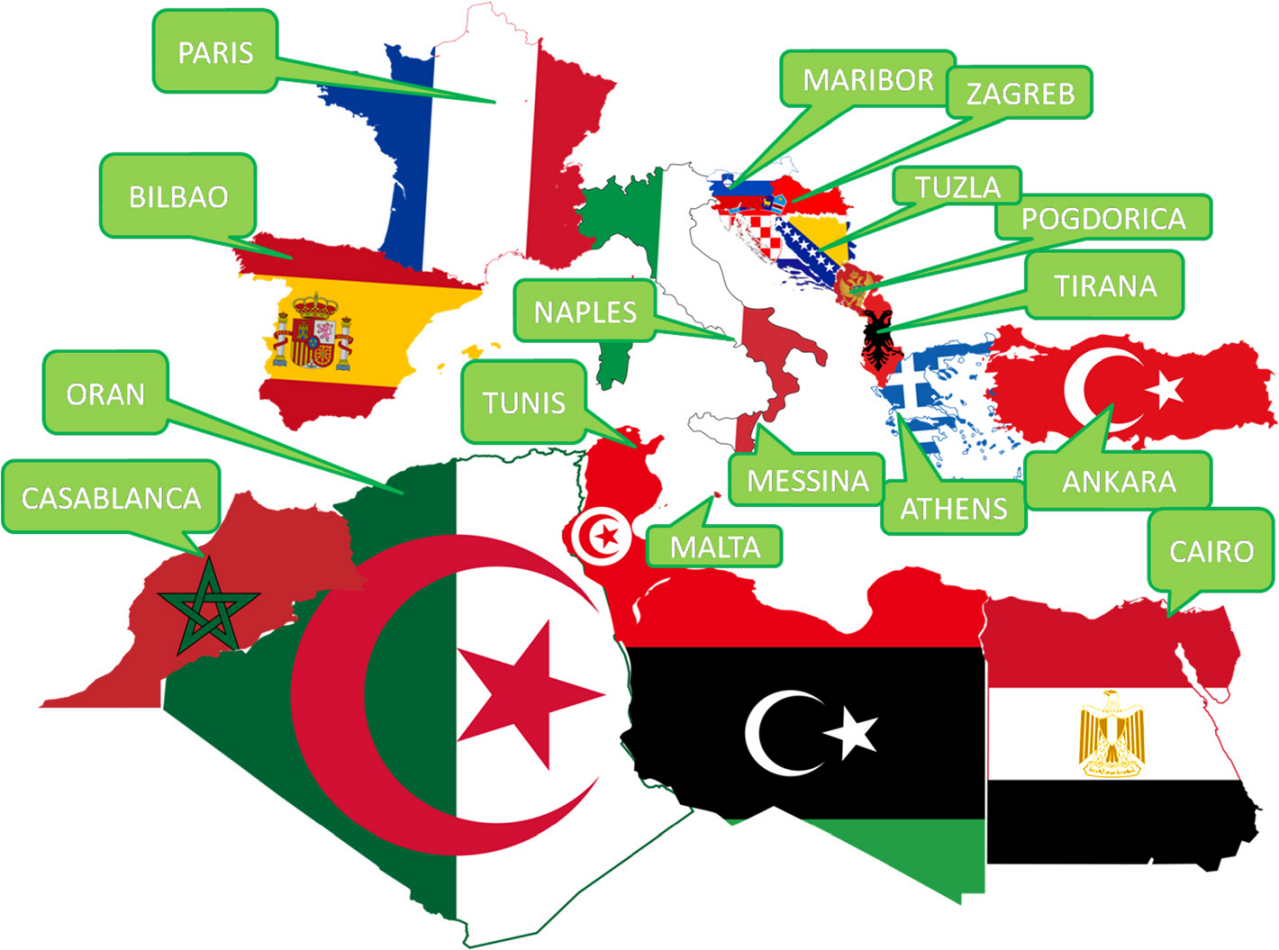
Map of referral centers in the participating countries.

Facilities and resources to control this modern non-communicable disease epidemic are limited in many of the less affluent countries of the world including some of the Mediterranean countries. The World Gastroenterology Organization (WGO) recognized that the ‘epidemic’ of CD cannot be met without considering the local resources: hence it recommended adapting the diagnostic protocol according to available resources [11]. Recently, European Society for Paediatric Gastroenterology, Hepatology and Nutrition (ESPGHAN) published new diagnostic criteria for CD to simplify CD diagnosis by avoiding small bowel biopsy in selected patients [12,13], namely patients with clear symptoms, tissue transglutaminase (tTG) levels 10-fold above the upper limit of normal, positive anti-endomysial antibodies (EMA) and specific HLA haplotype. This new algorithm, not requiring in all cases expensive endoscopy and pathology equipment, could facilitate the control over the coming CD epidemic in less privileged countries. The Mediterranean Network for the Management of Food-Induced Diseases (MEDICEL), which is an EUROMED-based action, to which all Mediterranean countries participate (Figure 1), aims at improving awareness of the forthcoming CD epidemic.

We aim to describe a cross-sectional picture of the clinical features and diagnostic facilities in 16 countries of the Mediterranean basin. Since the new ESPGHAN diagnostic protocol was recently published, our secondary aim is to estimate how many cases could be identified without a small intestinal biopsy in the same area.

## Methods

Unfortunately Syria, Lebanon and Libya did not participate to the network. Palestine was not able to comply with the requirement for the study. Israel could not obtain the ethical permission to use data already collected for a similar study.

Each referral center from Albania, Algeria, Bosnia Herzegovina, Croatia, Egypt, France, Greece, Italy - Messina and Naples - Malta, Montenegro, Morocco, Slovenia, Spain, Tunisia and Turkey provided a list of 50 unselected consecutive CD cases diagnosed in the center by the best locally available methodologies. Using a standardized form, the clinical and laboratory data leading to the diagnosis were collected for each case. The methods used in each center for the serum assay of tTG were recorded, together with their local normal values. We reviewed the pathology procedure used to evaluate small intestinal biopsy specimens and recorded the methods used to evaluate the HLA haplotype. EMA assay was available in 8/16 centers: due to the subjectivity of the method, we could not obtain a standardized comparable set of results.

According to the WGO diagnostic protocol, tTG are an acceptable proxy of the EMA antibodies, especially in countries with limited resources (“*The EMA test requires expert observers, and ELISA tests for detecting tTG antibodies should therefore be recommended in settings with low expertise”)*[11]. For these reasons, in the analysis, we did not considered the few available EMA values.

Based on the facilities and tests available in each center, for each case we estimated the probability of being diagnosed with CD by the new ESPGHAN diagnostic criteria alone, without small intestinal biopsy. For each case we summed up the number of diagnostic criteria met (clinical symptoms, tTG above 10 folds the upper limit of normal and specific HLA). The sum of the criteria determined the final diagnostic score, which thus ranged from 1 to 3. To reduce large tables, countries were often aggregated within their geographical region (Europe, Balkans, North Africa).

Data analysis was performed using the SPSS 16 software package (SPSS Inc., Chicago, IL, USA). Variables were log transformed when required because of their not normal distribution. Student’s t test and ANOVA were used to compare group means; non parametric tests were used when appropriate; p values <0.05 were considered significant.

### Ethics statement

Institutional review boards at each collection site approved the study in each country (Albania, Algeria, Bosnia Herzegovina, Croatia, Egypt, France, Greece, Italy, Malta, Montenegro, Morocco, Slovenia, Spain, Tunisia and Turkey) (Additional file 1).

## Results

A total of 800 forms were expected by the 16 CD referral centers of the Mediterranean Basin, 50 each, but 51 (6.3%) forms were excluded by the local centers because of missing data, thus 749 (93.7%) CD cases were included in the analysis.

### Clinical presentation

A total of 169/749 patients (22.5%) reported a case of CD among their first-degree relatives: mother in 44/749 (5.8%); father in 45/749 (6%); sibling in 45/749 (6%); and more than one relative in 35/749 (4.6%) cases. The female-to-male ratio was 1.6:1 (461 females; 288 males). Age at diagnosis ranged from 6 months to 18 years: 388 patients (51.8%) were diagnosed within the first 5 years of life, and 23.4% during the first two years of life. The mean age at diagnosis was 5.97 years (SD 4.35; median 5 years). The distribution of age at diagnosis did not differ significantly (ANOVA p >0,1) among the 3 geographical areas (Europe, Balkans and North Africa). Since the participants are referral centers, most patients had serious clinical complaints (Table 1). Clinical manifestations varied according to age (Table 2). Younger children (1-5 years old) more often suffered from diarrhea and food refusal than older children. Vomiting, food refusal and growth impairment were common across all ages. Older children and adolescents (>10 years) occasionally presented with extra-intestinal manifestations such as mood changes and anemia, but generally showed the same symptoms as younger children.

**Table 1:** Frequencies of symptoms in the three macro-areas

**Symptoms**		**Area**	
	**Europe**	**Balkans**	**Africa**
Chronic diarrhea	82 (35.3%)	149 (44.5%)	99 (54.4%)
Weight loss	81 (34.9%)	155 (46.2%)	67 (36.8%)
Food refusal	59 (25.4%)	107 (31.9%)	55 (30.2%)
Vomiting	122 (52.6%)	240 (71.6%)	113 (62.1%)
Anemia	22 (9.5%)	74 (22.1%)	31 (17.0%)
Constipation	11 (4.7%)	32 (9.5%)	8 (4.4%)
Other	13 (5.6%)	38 (11.3%)	18 (9.9%)
No symptoms	41 (17.7%)	3 (0.9%)	15 (8.3%)
Total	232	335	182

**Table 2:** Presenting symptoms by ages

**Symptoms**		**Age (years)**	
	**0-5**	**6-10**	**11-18**
Chronic diarrhea	188 (22.8%)	87 (17.9%)	55 (16.8%)
Weight loss	156 (18.9%)	78 (16%)	69 (21.1%)
Food refusal	128 (15.5%)	63 (13%)	30 (9.2%)
Vomiting	221 (26.8%)	159 (32.7%)	95 (29.1%)
Anemia	67 (8.1%)	37 (7.6%)	23 (7%)
Constipation	26 (3.2%)	18 (3.7%)	7 (2.1%)
Other symptoms	22 (2.7%)	22 (4.5%)	25 (7.6%)
No symptoms	15 (1.9%)	22 (4.5%)	23 (7%)
Total	824 (50.3%)	486 (29.7%)	327 (20%)

### Serology tests

tTG data were available for 748 cases. The cutoff values of normality in the various countries ranged between 7 and 20 UI/ml, and we normalized the reported raw values for the corresponding threshold of the center. The distribution of tTG values in times the normal local values shows that 252 (36.6%) patients had values 10 times the normal or more and a considerable number, 103 (13.7%), of CD cases were diagnosed by a tTG level not greater than twice the upper limit of the local normal values.

### Biopsy

706/749 (94.2%) CD patients underwent a duodenal biopsy. A biopsy report was not provided for 27/43 cases (62.8%) in Albania, for 8/42 cases (19%) in Bosnia Herzegovina, and for 4/36 cases (11.1%) in Egypt. Mild mucosal lesions (T0-T2) were found in 158 patients (22.4%), while 56 (7.9%) patients had partial atrophy (T3a), 492 (69.6%) patients had more severe atrophy (T3b and T3c).

### HLA typing

HLA typing was reported for all cases from Greece, Naples, Slovenia and Spain, in 14/42 of cases from Bosnia Herzegovina (33.3%), in 13/50 from Croatia (26%), 16/50 from Messina (Sicily) (22%) and 34/50 from Turkey (68%). HLA results were missing in countries of North Africa (Algeria, Morocco, Tunisia and Egypt), Albania and Montenegro. In conclusion, 368/749 CD cases were genotyped for HLA (49.1%). Of these 285 (77.4%) carry the DQ2 heterodimer, 76 (20%) the DQ8 heterodimer, whereas 7 (1.9%) do not carry either of these molecules.

### When could the biopsy be omitted?

Table 3 shows that 91.4% of cases had clear symptoms (criterion 1), 33.6% had x10 tTG titers (criterion 2) and 42.3% of patients underwent HLA typing (criterion 3). When we examined the sum of criteria for each individual, we found that 39% of patients had clinical symptoms only, 40% had two criteria, i.e., clinical symptoms plus serum tTGx10 normal or HLA, and only 154 (20.7%) of 749 patients had all 3 criteria thereby qualifying for a diagnosis without a biopsy. HLA typing was not available for half the cases; if we assume that all these cases had a compatible HLA, we may conclude that 304 (40.9%) cases would qualify for the biopsy-free diagnosis.

**Table 3:** Presence of the ESPGHAN diagnostic criteria in the 749 patients

**Symptoms**		**Frequency**	**Percentage**
	None	59	7.9
	Present	685	91.4
**tTG**			
	<10 times	496	66.2
	>10 times	252	33.6
**HLA**			
	Absent	432	57.7
	Present	317	42.3
**ESPGHAN diagnostic criteria**			
	1	289	38.5
	2	294	39.2
	3	154	20.5

**Figure 2:**
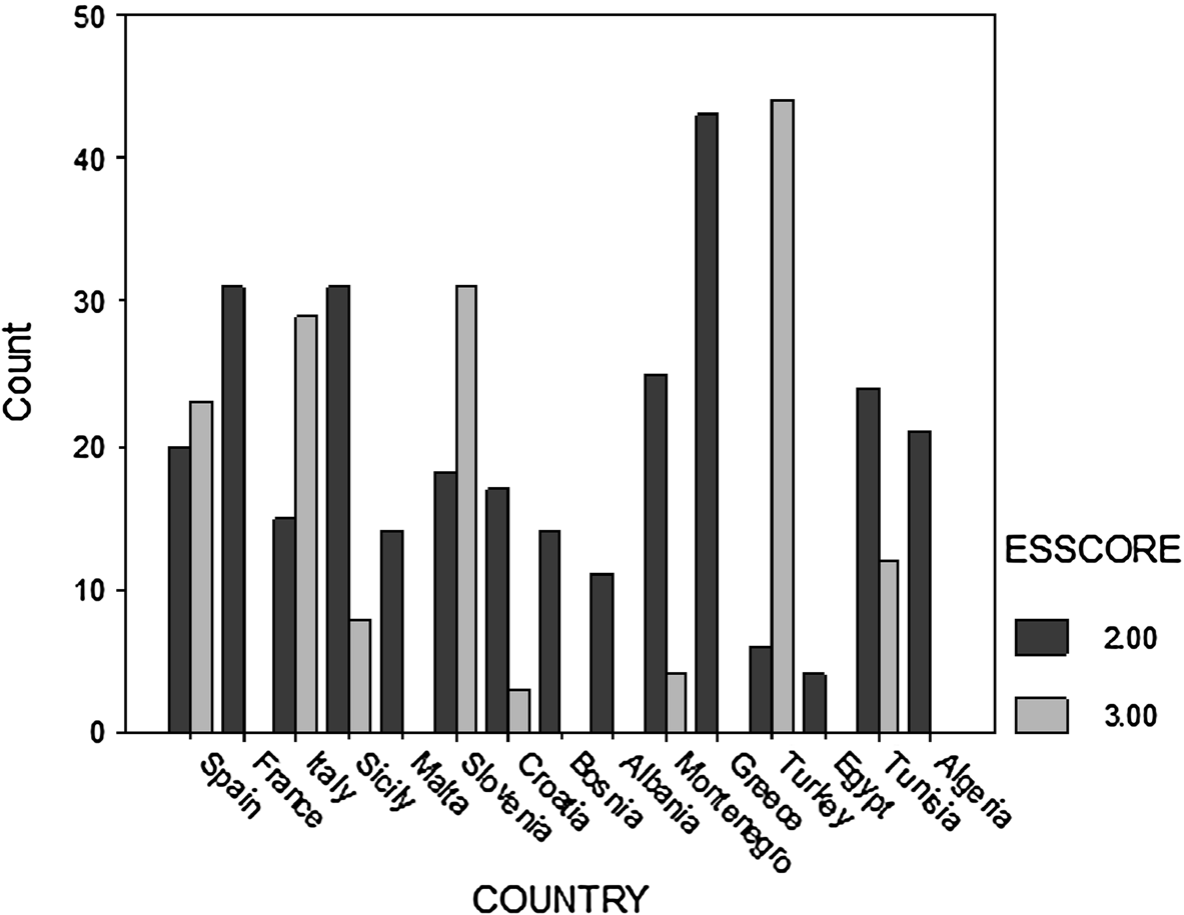
ESPGHAN score (ESSCORE) by country.

Figure 2 shows the percentage of cases with 2 and 3 criteria in the various countries. It is clear that for many countries (Albania, Algeria, Bosnia Herzegovina, Egypt, France, Greece, Malta and Morocco) not one case would qualify for the biopsy-free protocol. In fact, it would be applicable only for a sizeable number of cases from Italy, Slovenia, Spain, Tunisia and Turkey.

## Discussion

Given the worldwide increase in CD cases, there is an impelling need to simplify the diagnostic work-up to enable a timely diagnosis [11,12]. This is particularly true for countries that do not have access to sophisticated techniques. The WGO recommends evaluating the local availability of resources to develop a diagnostic protocol. We now provide a comprehensive picture of the diagnosis of CD in 16 Mediterranean countries. Being in European neighborhood area we evaluated whether the new ESPGHAN diagnostic protocol, which could potentially omit endoscopy and biopsy in a significant number of cases [13,14], could be applied efficiently in the 16 countries of the Mediterranean Basin who participate to the MEDICEL network. Indeed, in selected populations the “triple test” criteria appear to be helpful: a Spanish study [14] supports the view that in selected children who are symptomatic and positive for the triple test, CD diagnosis could be established independently of histological findings. Almost all cases here described were diagnosed by a small intestinal biopsy, while the aim of the new ESPGHAN criteria is to reduce the requirement for such and invasive procedures. We shared among the 16 partners the importance of avoiding, at least in a percentage of cases, such an invasive technique.

Unfortunately, in our setting only 20% of suspected CD cases with clear clinical symptoms might qualify for a CD diagnosis without a small intestinal biopsy. This percentage would have increased to about 40% if HLA testing had been available. This limitation is not confined to less equipped countries, but it also applies to many European centers with a long experience in CD diagnosis.

**Figure 3:**
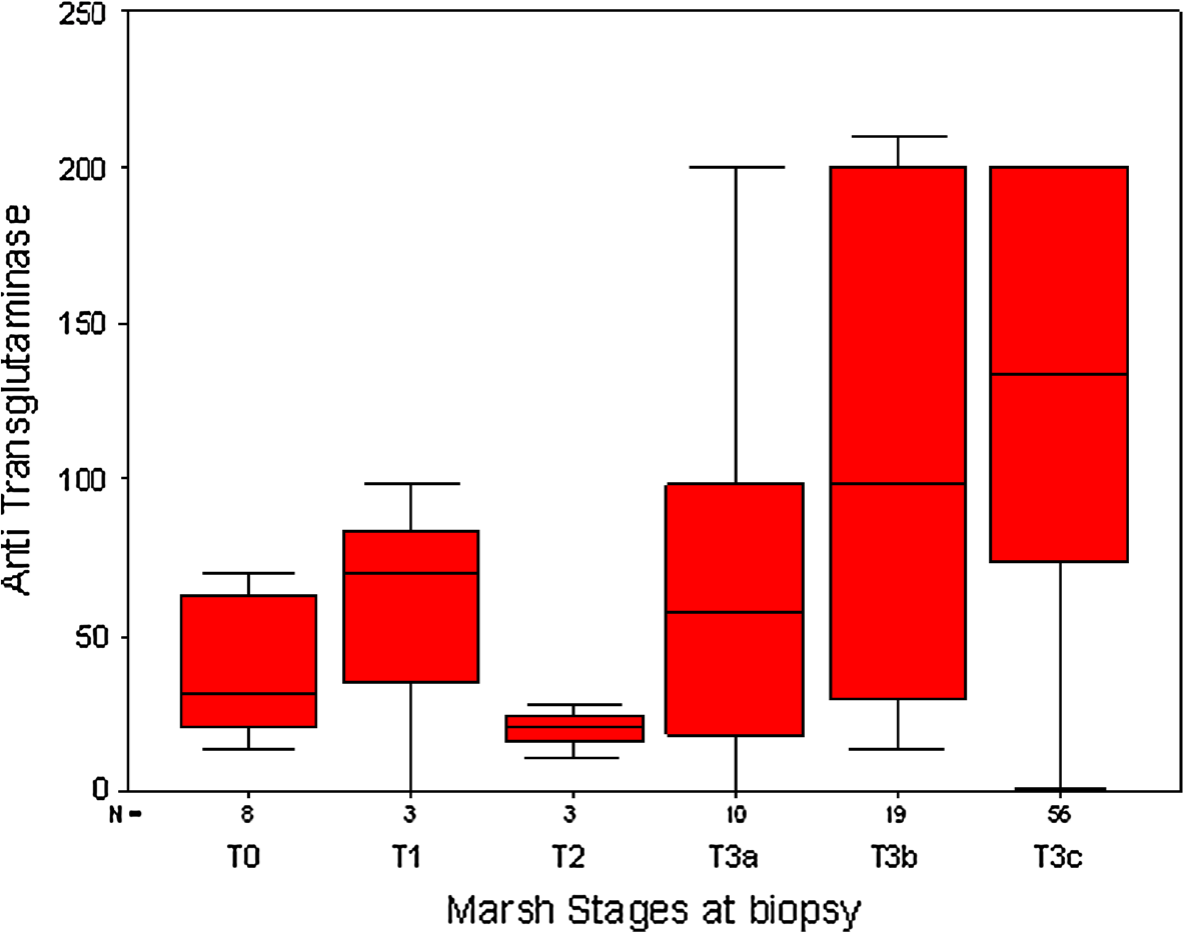
Median tTG levels by Marsh grading at biopsy (Messina and Naples, Italy).

The chance of avoiding a small intestinal biopsy is based on the strength of the correlation between the tTG and the stage of mucosal damage. Recently Wakim-Fleming J et al. [15] suggested that titres of IgA tTG > 118 IU identified patients with CD with a 2% false-positive rate. Titres of 21-118 UI, in combination with EMA dilution titer ≥1:160, had a positive predictive value of 83% for CD. Popp et al. [16] also found that in diabetic patients high tTG titres predict a diagnosis of CD, when associated to EMA positivity. The relationship between antibodies and biopsy is crucial: we show on Table 4 the Spearman Correlation Coefficients between the values of the tTG(log) and the Marsh stages at biopsy in each country. It is clear that the correlation is strong and significant only in Italy (Messina and Naples), while it is not significant in all other countries. The basic assumption that tTG antibodies predict severe mucosal damage is not confirmed in this set of data. The shape of the correlation in Italy (Messina and Naples) shows that tTG does increase with Marsh Stages (Spearman Rho?=?0.44), but there are very ample 95% Confidence Intervals around the median at all Marsh stages, making the basic assumption quite fragile also in this country (Figure 3). Based on our data, tTG does not appear to be actually a proxy of the degree of mucosal damage as evaluated by the Marsh score. Also in Italy, where there is a clear correlation, the interquartile range is very large, making this correlation unsafe to predict the mucosal damage by the high levels of antibody production. Although the correlation between the mucosal damage, estimated by the Marsh stages, and the level of tTG antibodies has been confirmed in several relevant studies [13], this correlation has several weakness, due to the nature of the data. tTG antibodies, as all antibodies, do not have a normal distribution, but a logarithm distribution and Marsh stages have an ordinal distribution, not a continuous one. So the relationship between these two variables is intrinsically prone to large or very large confidence intervals, which reflect not only the nature of the variables, but also the wide polymorphism of the phenotype of the disease. The quality of serological test and the scarcity of HLA testing are the critical points that, at present, limit the chance to diagnose CD without a biopsy.

**Table 4:** Correlation between tTG and Marsh stages

**Country**	**Spearman Rho**	**p value**
Albania	0.42	0.01
Algeria	-0.14	0.32
Bosnia	0.09	0.58
Croatia	-0.07	0.6
Egypt	0.17	0.3
France	0.06	0.6
Greece	0.007	0.9
Malta	0.18	0.9
Montenegro	0.25	0.079
Morocco	0.33	0.018
Naples	0.53	0.0001
Sicily	0.37	0.008
Slovenia	0.13	0.33
Spain	-0.09	0.5
Tunisia	0.19	0.17
Turkey	0.21	0.16
**Total**	0.12	0.002

Unfortunately, in several of the participating referral centers, the laboratory experience to assay the tTG is limited and performed only when financial resources are available. Some laboratories produce semi-quantitative data, increasing the uncertainty of the assessment. One of the objectives of the MEDICEL network is to support the upgrading of local diagnostic resources: hence we are now running *ad hoc* procedure to standardize the method of antibody assay and to increase the availability of HLA haplotyping by exploiting the new technologies that attempt to bring the test to the point of care [17,18].

This cross-sectional study provides the first picture of the profile of CD in the countries studied and of the diagnostic resources available in the referral centers. Most cases were symptomatic showing the classical clinical profile. This will probably change significantly in the near future when awareness about CD increases, as has occurred in several European countries [3,10]. This study suffers from the objective limitation of being a retrospective study. Nevertheless, we needed a cross-sectional picture of the pattern of celiac disease in the area, for which this kind of study is rapidly informative. In the same area we have already started a prospective study in order to validate the findings of this actual study.

## Conclusion

In conclusion, this cross-sectional survey provides a multifaceted picture of the CD domain in the Mediterranean area. Being aware of the expanding epidemic of CD over the wheat consuming populations, we hope that simplified diagnostic criteria, possibly avoiding the expensive biopsy, could help to diagnose cases outside the very few referral centers in developing countries. Unfortunately this study does not support this chance to date, but does identify the critical points to be met in order to expand the advantages of the new ESPGHAN diagnostic protocol, especially in countries that need this change the most. These results provide to each participant country required data to develop local strategies according to the WGO recommendations.

## Competing interests

The authors declare that they have no competing interests.

## Authors’ contributions

FT, LA, RA and LG planned the study, developed the forms, run the analysis and lead the writing of the manuscript. PR, MBH, AK, SK, GM, ZM made substantial contributions to conception and design and acquisition of data. AA, MAZ, JRB, GB, SB, SC, VD, JPH, II, DMT, ER, ST, VV, CA have been involved in drafting the manuscript and collecting data. All authors read and approved the final manuscript.

## Pre-publication history

The pre-publication history for this paper can be accessed here:

http://www.biomedcentral.com/1471-230X/14/24/prepub

## Supplementary Material

Additional file 1 National Ethical Permissions. Click here for file
